# Intersections in Neuropsychiatric and Metabolic Disorders: Possible Role of TRPA1 Channels

**DOI:** 10.3389/fendo.2021.771575

**Published:** 2021-11-29

**Authors:** Rupinder Kaur Sodhi, Raghunath Singh, Yashika Bansal, Mahendra Bishnoi, Ishwar Parhar, Anurag Kuhad, Tomoko Soga

**Affiliations:** ^1^ Pharmacology Research Laboratory, University Institute of Pharmaceutical Sciences, University Grants Commission, Center of Advanced Studies (UGC-CAS), Panjab University, Chandigarh, India; ^2^ Schizophrenia Division, Centre for Addiction and Mental Health (CAMH), Toronto, ON, Canada; ^3^ Campbell Family Mental Health Research Institute, Centre for Addiction and Mental Health (CAMH), Toronto, ON, Canada; ^4^ TR(i)P for Health Laboratory, Centre of Excellence in Functional Foods, Department of Food and Nutritional Biotechnology, National Agri-Food Biotechnology Institute (NABI), Punjab, India; ^5^ Brain Research Institute Monash Sunway (BRIMS), Jeffrey Cheah School of Medicine and Health Science, Monash University Malaysia, Bandar Sunway, Malaysia

**Keywords:** TRPA1, Neuropsychiatric disorders, metabolic disorders, appetite control, insulin resistance, obesity

## Abstract

Neuropsychiatric disorders (NPDs) are a huge burden to the patient, their family, and society. NPDs have been greatly associated with cardio-metabolic comorbidities such as obesity, type-2 diabetes mellitus, dysglycaemia, insulin resistance, dyslipidemia, atherosclerosis, and other cardiovascular disorders. Antipsychotics, which are frontline drugs in the treatment of schizophrenia and off-label use in other NPDs, also add to this burden by causing severe metabolic perturbations. Despite decades of research, the mechanism deciphering the link between neuropsychiatric and metabolic disorders is still unclear. In recent years, transient receptor potential Ankyrin 1 (TRPA1) channel has emerged as a potential therapeutic target for modulators. TRPA1 agonists/antagonists have shown efficacy in both neuropsychiatric disorders and appetite regulation and thus provide a crucial link between both. TRPA1 channels are activated by compounds such as cinnamaldehyde, allyl isothiocyanate, allicin and methyl syringate, which are present naturally in food items such as cinnamon, wasabi, mustard, garlic, etc. As these are present in many daily food items, it could also improve patient compliance and reduce the patients’ monetary burden. In this review, we have tried to present evidence of the possible involvement of TRPA1 channels in neuropsychiatric and metabolic disorders and a possible hint towards using TRPA1 modulators to target appetite, lipid metabolism, glucose and insulin homeostasis and inflammation associated with NPDs.

## Introduction

Neuropsychiatric disorders (NPDs) such as depression, dementia, psychosis, and bipolar disorder prompt an unhealthy lifestyle (such as cigarette smoking, alcohol consumption, altered eating habits and physical inactivity), leading to obesity. Schizophrenic patients have shown a higher prevalence of obesity than the general population (1). Obesity and NPDs are linked to each other bidirectionally. On one hand, obesity makes a person more vulnerable to anxiety and depression-like behavior and on the other hand presence of any of the NPDs further enhances the risk of obesity in non-obese patients (2–4).

Association of NPDs with diabetes, obesity or insulin resistance has been an area of interest of researchers for a long time, and the very first reports of metabolic disorders associated with psychiatric disorders trace back to nearly four centuries ago in the 1600s by Thomas Willis (5). He suggested that diabetes is mainly precipitated in people undergoing mental stress such as grief, sorrow and sadness. Further reports assert the notion that insanity and diabetes are frequently co-expressed in patients/families (6). The predominance rates for type 2 diabetes mellitus and insulin irregularities are roughly sevenfold higher in Huntington’s disease patients when compared to healthy controls (7). Also, clinical explorations in intrinsic neurodegenerative disorders advocate that over 20% of those influenced will end up having metabolic comorbidities such as insulin resistance/obesity (8). Type 2 diabetes mellitus (T2DM) is a self-determining risk factor for dementia, with the diabetic population being twofold more susceptible to dementia (9). A study reported that about 45% of patients with mental health disorders are comorbid with obesity and metabolic syndrome (10).

Similarly, patients suffering from attention deficit hyperactivity disorder, regardless of their age, are vulnerable to obesity and likewise, this disorder is more prevalent in obese teenagers (11). Patients with bipolar disorder are the ones who have the highest incidences of obesity and other metabolic disturbances (12). Adding to this, antipsychotics (APs), which are used to treat schizophrenia and bipolar disorder, are also responsible for diabetes and metabolic syndrome precipitation. Another recent clinical study stated that out of the total participants for each disorder, nearly 47.5% with bipolar disorder, 38.4% with anxiety and 39% with major depression had metabolic syndrome (13). APs, mainly the atypical ones, which are being used enormously during the past two decades, are one of the major causes of metabolic perturbations. AP-induced metabolic alterations (AIMA), including appetite dysregulation, obesity, dyslipidemia, T2DM, insulin resistance, and cardiovascular diseases lead to decreased life expectancy and poor patient compliance (14, 15). It was reported that about 30% of diabetes/insulin resistance cases are due to AP use in the psychiatric population (16). APs have an affinity towards multiple neurotransmitter-receptors (mainly serotonergic, histaminergic, dopaminergic and cholinergic receptors), leading to a wide variety of desirable and undesirable effects (17). There is humongous literature available affirming that atypical APs, upregulate the appetite-inducing peptides [neuropeptide Y (NPY), agouti-related peptide (AgRP), orexins, ghrelin and melanin-concentrating hormone (MCH)] while downregulating the anorexigenic peptides [pro-opiomelanocortin (POMC), cocaine- and amphetamine-regulated transcript (CART), α-melanocyte-stimulating hormone (α-MSH), pancreatic polypeptide, peptide YY, glucagon-like peptide-1 (GLP-1), insulin, cholecystokinin (CCK), and glucose-dependent insulinotropic peptide (GIP)]. Altered appetite regulation disturbs the energy balance, apparently causing obesity and metabolic syndrome (18, 19).

Despite the availability of diverse pharmacological treatment options to control obesity and maintain energy homeostasis, there is a pressing need to find other pharmacological/non-pharmacological treatment options with fewer side effects. Numerous receptor pathways, hormones, peptides and neurotransmitters are linked to regulating food intake and energy homeostasis, which could have a potential uncovered role in controlling metabolic syndrome. One such receptor system is the Transient Receptor Potential (TRP) system. There are various subtypes of TRP channels that play a pivotal role in food reward, energy intake and expenditure, metabolism of glucose and fats, several vital biological functions and they are primarily involved in the influx of cations such as Na^+^ and Ca^2+^.

TRP channel family comprises about 30 different subtypes of plasma membrane ion channels which are broadly divided into two groups: Group 1 consisting of TRPV (Vanilloid), TRPC (Canonical), TRPM (Melastatin), TRPN (No mechanoreceptor potential) and TRPA (Ankyrin) while Group 2 consists of TRPP (Polycystic) and TRPML (Mucolipin). These different subtypes are involved in function as well as respond to a wide variety of stimuli such as hot and cold sensitivity, itch, respiration, pain, mechanical or chemical stimulation, oxidative stress, or light. TRPV channels have already shown a potential role in controlling appetite, energy balance and lipid homeostasis in normal physiology (20) and AIMA (21, 22). TRP channels, by modulating cellular Ca^2+^ levels are involved in the etiology of NPDs such as anxiety, fear and bipolar disorder. For example: - Riccio et al. reported that TRPC5 had an important responsibility in fear induction and attenuates to conditioned fear under certain conditions. They also concluded that deletion of TRPC4 was responsible an anxiolytic-like behavioral phenotype and normal expression of the TRPC4 subunit in brain circuits might be required for behavioral responses in anxiety-inducing stimuli (23). TRPM2 was also found to be involved in bipolar disorder pathogenesis (24). An important subtype of the TRP channel receptor, i.e. TRPA, has also evolved to show potential in NPD pathophysiology and food reward modulation along with various metabolic functions. TRPA1 channels are involved in both metabolic regulation (25) as well as neuropsychiatric complications. In this review, we try to discuss the potential of TRPA1 channels in controlling food intake and energy homeostasis and ultimately NPDs.

## TRPA1 Channels in Brain

TRPA1 are the most comprehensively studied TRPA channels among the seven TRPA subfamilies. It contains 14 N-terminal ankyrin repeats and mainly functions as a temperature sensor, i.e. responds to hot/cold temperatures; however, its sensitivity varies in different species. It also responds to pain, itch and is activated by various common chemicals present in food, cosmetics and environmental pollutants (26–28). TRPA1 channels are localized in diverse parts of the brain, such as nociceptive primary afferent sensory neurons present in the trigeminal ganglia (29), hippocampus, cortex, supraoptic nucleus of the brain stem and layers of rodent somatosensory cortex neurons (30). TRPA1 activation in the hippocampal astrocytes leads to an increase in extracellular GABA along with activation of cannabinoid receptors. This increase in GABA hauls down the efficacy of inhibitory synapses between the interneurons. TRPA1 channels are also highly expressed in the endothelium and cerebral arteries, and their activation helps in protection against stroke or hypoxic damage (31). TRPA1 channels are closely related to TRPV1 channels in localization and functions. 97% of TRPA1-expressing sensory neurons express TRPV1 channels, whereas 30% of TRPV1-expressing neurons express TRPA1 (32) and this co-expression could form the molecular basis of nociception in chronic abnormal pain induced by inflammation.

## TRPA1 Channels and NPDs

Studies have revealed the involvement of TRPA1 channel modulation in the pathogenesis or treatment of various NPDs such as Alzheimer’s, depression, anxiety (33, 34). TRPA1-channel function inhibition has been shown to lessen the behavioral dysfunction, Aβ plaque deposition and neuroinflammation in APP/PS1 Tg mice brain (35). TRPA1 channels also play a crucial role in synapse efficacy by modulating resting Ca^2+^ in astrocytes (36). Activation of astrocytic TRPA1 channels increases the Ca^2+^ hyperactivity in the hippocampus similar to Aβ oligomers, and TRPA1 channel inhibition is protective against Aβ-induced early synaptic dysregulation (37). Quantitative structure-activity relationship (QSAR) and molecular docking studies have recently reported that the use of TRPA1 inhibitors could be an important treatment strategy for multiple sclerosis (38). Supporting this, a study in TRPA1^-/-^ mice showed that TRPA1 deficiency is defensive in cuprizone-induced demyelination (39). TRPA1 channel ligands also modulate its binding to the σ1R receptor to modulate the cannabinoid pathway in the brain (40). Likewise, ligands such as AM630 and AM251, which are cannabinoid receptor antagonists, tend to activate TRPA1 by promoting the accumulation of Ca^2+^ in the trigeminal neurons. This modulation protected the animals against capsaicin-induced hyperalgesia in wild type mice but not in TRPA1^-/-^ mice, therefore, confirming the role of TRPA1 channels in capsaicin-induced thermal hyperalgesia (41). Further, a study showing higher mRNA expression of TRPA1 in the posterior hypothalamus of normotensive as well as hypertensive rats suggested its role in hypertension pathology (42). Imperative pathways involved in the inflammatory response, i.e. Prostaglandin E2 (PGE2) and bradykinin (BK) signaling, also activate TRPA1 channels *via* PKCϵ cascade. Furthermore, mGluR5 signaling results in thermal and mechanical hyperalgesia due to sensitization of both TRPA1 and TRPV1 channels (43).

TRPA1 channels have also been recounted as an underlying cause of cortical spreading depression (CSD), which further acts as a base of migraine pain. Activation of TRPA1 channels triggers migraine, while its desensitization helps in reducing migraine pain (44, 45). A study in TRPA1 deficient mice by Nassini et al. (2012) reported that umbellulone (a TRPA1 agonist) caused nocioceptive behavior after stimulation of trigeminal nerve terminals in wild-type mice, but not in TRPA1 deficient mice (46). Jiang et al. (2018) have reported that umbellulone, aids CSD propagation to a longer distance in mice cortical brain slices. Further, TRPA1 antagonists such as HC-030031 and A-967079 suppressed CSD by increasing the CSD latency. They also reported that allyl isothiocyanate (AITC) application reversed the suppression of CSD by HC-030031. However, no significant reversal was seen in the case of A-967079 (47). Another study, done by the same group aimed to explore the probable mechanism of TRPA1 involvement in depression, revealed that intracerebroventricular (ICV) perfusion of TRPA1 antibody in rats led to inhibition of CSD. It was further co-related to a lower level of oxidative stress as evidenced by reduced malondialdehyde levels in the cortex. Further investigations revealed that reactive oxygen species (ROS) also lead to the expedition of CSD in mouse cortical brain slices while ROS inhibitor, tempol, significantly extended the latency of CSD. Umbellulone, on the other hand, reversed the CSD suppression produced by ROS inhibitor and both ROS and TRPA1 activation overturned the decreased cortical susceptibility to CSD by the anti-CGRP (calcitonin gene-related peptide) antibody, indicating that ROS/TRPA1/CGRP signaling regulates cortical susceptibility to CSD leading to migraine (48).

TRPA1 channel inhibition by ICV (30 nmol in 2 μL) as well as oral administration of HC-030031 (100 mg/kg) also produces antidepressant and anxiolytic-like actions in mice as evidenced by reduced immobility time in the forced swim test and increased open arms exploration in the elevated plus-maze test. These effects were reversed by TRPA1 agonist cinnamaldehyde pretreatment; however, it was ineffective as *per se*. Likewise, TRPA1^-/-^ mice also possess antidepressant-like and anxiolytic phenotypes when tested for immobility and anxiety in a forced swim test and an elevated plus-maze test. TRPA1 antagonism, as well as gene deletion, didn’t show any effect on locomotor activity (34). TRPA1 channels are also involved in innate fear responses aroused by their predator’s odor (such as snake skin) in mice. TRPA1 inactivation reduced innate defensive behavior evoked by snake skin and TRPA1–expressing trigeminal neurons plays a key part in 2-methyl butyrate-evoked innate freezing behavior. TRPA1^-/-^ mice do not show 2-methyl butyrate-evoked innate freezing responses. The study also concluded that innate fear/defensive behaviors, evoked by predator odor are regulated by TRPA1-mediated nociception (49).

Literature supports the possible involvement of TRP channels in schizophrenia (50); therefore, further studies investigating the role of specific subtypes of TRP channels in schizophrenia linked to obesity and metabolic disorder could help find potential candidates to tackle the problem.

## TRPA1 Channels in Metabolic Disorders

### TRPA1 in Obesity, Glucose and Insulin Homeostasis

Peripherally, TRPA1 channels are distributed in various organs such as gastrointestinal tract (GIT), pancreas, myenteric plexus, duodenal epithelial cells, bladder, keratinocytes, dorsal root ganglion and are therefore thought to facilitate secretory function. Supporting this statement, many studies have shown TRPA1 channels to be involved in various gastric and metabolic processes such as ghrelin release, changes in blood glucose levels, appetite control, GLP-1 regulation, gastric emptying and others (51–54). Elevated GLP-1 has been reported after AITC treatment in the GLUTag cells in a response similar to as shown by polyunsaturated fatty acids (PUFAs) (53).

TRPA1 channels, present in the rat pancreatic β-cells and rat pancreatic beta-cell line (RINm5F), facilitate the dose-dependent release of insulin after AITC treatment, similar to glucose treatment. These effects of AITC were diminished in the presence of HC-030031. The study also confirmed TRPA1-mediated enhancement in insulin release in the presence of tetrodotoxin (a voltage-dependent Na^+^ channel blocker) and nimodipine (a voltage-dependent Ca^2+^ channel blocker) (55). The antidiabetic and anti-obesity effects of TRPA1 agonists have been well reported. Cinnamaldehyde has been used in traditional medicine for its antidiabetic effects as it is known to enhance glucose uptake and increase expression of glucose transporter GLUT-1 (56), decrease glycosylated hemoglobin, serum triglyceride and cholesterol levels, increase high-density lipoprotein, insulin and hepatic glycogen (57). It also prevents lipid accretion, fasting-induced hyperphagia, visceral fat deposition and alterations in the levels of leptin and ghrelin caused by a high-fat diet (58, 59). It reduces ghrelin secretion in conjunction with improving insulin sensitivity (60). These antidiabetic effects of cinnamaldehyde were also seen in patients with T2DM receiving hypoglycemic agents. Cinnamaldehyde supplementation was able to significantly reduce glycated hemoglobin, mean systolic and diastolic blood pressure, fasting plasma glucose, and body mass index over the period of 12 weeks (61). Cinnamaldehyde also improves glucose tolerance as seen in oral glucose tolerance test after acute treatment in healthy people (62). Collectively, this evidence suggests a strong involvement of TRPA1 agonist cinnamaldehyde in controlling appetite and being a promising candidate to manage diabetes and obesity. Not just cinnamaldehyde, AITC has also shown prominent effects as a potential antidiabetic compound as it helps in improving insulin resistance, improves glucose intake, mitochondrial activity, glycosylated hemoglobin, GLUT-4 translocation to the membrane from the cytoplasm and protects the animals from high-fat diet-induced hepatic steatosis, body weight gain and lipid dysregulation (51). It is important to note that TRPA1 modulators not only regulate glucose and insulin homeostasis but also control insulin release from the pancreatic β-cells. AITC, hydrogen peroxide, 4-hydroxynonenal and prostaglandin J2 induced depolarization, Ca^2+^ influx and further insulin release from pancreatic β- cell lines (55) and RINm5F cells (63). This insulin release was blocked by HC-030031 and AP-18 indicating that TRPA1 channels play an imperative role in insulin secretion from the pancreas.

The involvement of TRPA1 in pain perception makes it a potential therapeutic target for the treatment of other comorbid conditions associated with diabetes. TRPA1 antagonists have shown reduced diabetic neuropathy development in animals, but detailed studies are still required (64, 65). Another interesting study has reported reduced TRPA1 channel function in the DRG neurons upon activation of AMP-activated protein kinase (AMPK). The study revealed that metformin (an AMPK activator) inhibits TRPA1 activity by means of diminishing the amount of membrane-associated TRPA1. AMPK plays a significant role in energy homeostasis, and its activation stimulates hepatic fatty acid oxidation, inhibition of cholesterol synthesis, increased glucose uptake, along with other regulatory effects. But this inhibition of TRPA1 activity by AMPK activation was seen to be a helpful factor in preventing diabetic neuropathy as it led to the deterrence of mechanical allodynia in diabetic mice (66).

### TRPA1 in Lipid Homeostasis

TRPA1 channels are also involved in atherogenesis, a study conducted in 2016 has shown that apoE^-/-^ mice show higher TRPA1 channel expression in aortas than wild type mice (67). The study also revealed that genetic deletion of TRPA1 exacerbated the atherosclerotic lesion in apoE^-/-^ mice, whereas treatment with AITC reduced the atherosclerotic lesion in apoE^-/-^ mice but not in apoE^-/-^TRPA1^-/-^ mice, suggesting its involvement in atherosclerosis development. TRPA1 inhibition by HC-030031 also led to lipid accumulation. Another study conducted in HEK293 cell lines and TRPA1 null mice showed that PUFAs activate mammalian TRPA1, and TRPA1 is needed for PUFAs to stimulate enteroendocrine cells and sensory neurons to elicit cholecystokinin (CCK) secretion (68). Another study reported that TRPA1 channels get activated in response to bradykinin, diacylglycerol and arachidonic acid, although the mechanism is not fully illustrated yet (69). Cinnamaldehyde in the form of cinnamon powder has also been shown to reduce glucose, total cholesterol, triglycerides, and LDL cholesterol levels in T2DM patients (70). Furthermore, cinnamaldehyde treatment reduced the serum levels of triglycerides, total cholesterol, c-LDL and elevated the c-HDL levels in high-fat diet rats (71). This evidence suggests the involvement of TRPA1 in lipid homeostasis. Additionally, a study in 3T3-L1 cells hinted towards TRPA1 activation as a potential mechanism for the anti-lipid accumulation effect of *trans*-pellitorine (72). Literature also points at the involvement of TRPA1 channels in regulating uncoupling protein 1 (UCP1) expression. Oleuropein aglycone present in extra virgin olive oil was found to activate both TRPA1 and TRPV1. Also, it enhanced the UCP1 expression in intrascapular brown adipose tissue by stimulating noradrenaline secretion *via* the β2- and β3-adrenoceptors following TRPA1 and TRPV1 activation and reduced the visceral fat content in high fat diet-fed rats (73).

### TRPA1 in Adipokine and Cytokine Regulation

Other than their involvement in lipid and insulin homeostasis, studies involving TRPA1 agonists suggest that TRPA1 channels are also involved in adipokine-cytokine (adiponectin, leptin, TNF- α and IL-6) regulation. Adiponectin is essential for fatty acid breakdown and is an anti-inflammatory adipokine, which is decreased in T2DM (74) and obese patients (75). In other words, adiponectin levels are inversely proportional to insulin resistance. Cinnamaldehyde treatment increases adiponectin levels while also maintaining lipid homeostasis in rats (76) as well as adipocytes (77). Leptin, another major hormone involved in controlling appetite, releases from the adipocytes. It directly binds to its receptors and mediates energy expenditure, reduces food intake, affects insulin signaling and growth hormone. Leptin receptors are present peripherally on adipocytes and centrally in the hypothalamus and hippocampus. Several evidences of dysfunction in leptin signaling have been identified in diabetes, obesity, and metabolic disorders (78, 79). Hyperphagia and weight gain are typical characteristics of leptin deficiency; however, leptin levels are seen to be increased in some cases of obesity and diabetes owing to leptin resistance. Interference in leptin signaling in the form of leptin resistance has also been seen with antipsychotic drugs such as olanzapine used to treat psychosis and other neuropsychiatric disorders (80). Cinnamaldehyde decreases the elevated leptin levels in high-fat diet-fed mice (58, 81). Further, inflammation is an essential factor in the pathogenesis of insulin resistance. Studies have demonstrated interlinking between inflammation and insulin resistance as well as obesity as one leading to another and vice versa. Obesity and insulin resistance are involved in producing low-grade inflammation in the adipose tissue, leading to systemic and local insulin resistance (82). Cytokines such as TNF-α, IL-6 and IL-1β are responsible for producing insulin resistance by reducing insulin secretion from the pancreatic β-cells and reducing the insulin utilizing ability of cells (83–86). Cinnamaldehyde decreases the mRNA expression of TNF-α in adipose tissue in mice (87) and has also shown anti-inflammatory properties in response to LPS-induced inflammation in mice by decreasing TNF-α, IL-1β and interferon-gamma (IFN-γ) levels in mice (88). AITC as well possesses anti-inflammatory potential as demonstrated by reduced mRNA expression of TNF-α, IL-1β and enhanced gene expression of the anti-inflammatory, antioxidant heme oxygenase-1 (HO-1) *in vivo* as well as *in vitro* (89). However, none of these studies has directly co-related these effects with TRPA1 channel activation, but as they all involve specific TRPA1 agonists, future studies are envisaged indicating direct relation.

## TRPA1 Channels in Appetite Regulation

### TRPA1 in Peripheral Appetite-Regulating Hormones

The existence of TRPA1 channels in the enterochromaffin cells in the small intestine (specifically duodenum and jejunum) and cells expressing CCK and serotonin indicates its crucial role in controlling appetite as these both are major regulators of hunger in the GIT (90). Asserting this statement, a study in STC-1 cells showed increased CCK release upon AITC (91), naringenin (92) and hesperetin treatment (93) *via* stimulation of TRPA1 channels. This release was reduced upon ruthenium red or HC-030031 treatment. Additionally, this enhanced release was absent in the presence of L-type calcium channel blocker, which means that it is interwoven with intracellular calcium levels (91). Similarly, it was observed that inhibition of TRPA1 channels by HC-030031 suppresses aldehyde-induced CCK secretion in STC-1 cells (94). Cinnamaldehyde treatment decreased ghrelin secretion and gastric emptying rate in mice and also helped in reducing body weight in high-fat diet-induced obesity in mice (95). It also helped in normalizing the blood glucose levels as observed in the oral glucose tolerance test. It also increased the levels of acyl-CoA synthetase 4, an enzyme aiding in β-oxidation of fatty acids. However, recently there have been contradicting studies that have shown TRPA1 agonism to be responsible for enhanced contractions in the lower GIT and thus increases GIT motility (96). TRPA1 agonists have also been shown to play a role in adipogenesis. Oral cinnamaldehyde treatment helped in reducing mesenteric adipose tissue while upregulating expression of different GLUTs and carnitine palmitoyltransferase 1A (responsible for fatty acid oxidation) in the white adipose tissue (52).

In addition to the presence in the small intestine, TRPA1 channels are also present in the colon. Their presence in the colon was confirmed by *in-situ* hybridization and immunohistochemistry in the surface epithelium of the colon in rats. As described earlier, activation of TRPA1 channels induces PGE_2_ activation leading to anion secretion in the epithelium of the colon. It was reported that TRPA1 activation impedes spontaneous contractions and transit by directly stimulating myenteric neurons. Therefore, TRPA1 may also play a role in washing out harmful compounds and preserving gut microbiota, thus helping relieve constipation (97). Also, TRPA1 activators such as carvacryl acetate are known to decrease intestinal mucositis in mice (98). A healthy gut is essential in conserving normal appetite and metabolic functions; these protective effects of TRPA1 channels also serve as an added advantage.

TRPA1 channels play a significant role in taste perception. As we know, TRPA1 channels are co-expressed with TRPV1 channels in trigeminal nerves, which are present in hefty volumes in the taste cells along with vagus and glossopharyngeal nerve (99). Because these channels are activated by many chemicals such as allicin (garlic), isothiocyanates (wasabi, horseradish), curcumin (turmeric), eugenol (clove), thymol, cinnamaldehyde; activation of these channels by these food components helps in taste perception (28). The food substances that are irritable in taste are mostly felt because of TRPA1 channel activation (100). TRPA1 channel agonists cinnamaldehyde, methyl syringate and AITC, showed abridged food consumption by delaying gastric emptying (101). On the other hand, these effects were seen to be absent in animals treated with TRPA1 antagonist HC-030031 or ruthenium red. Food intake regulation measured by the levels of plasma PYY and GLP-1 also demonstrated elevated plasma PYY by cinnamaldehyde and methyl syringate but not in the presence of HC-030031 and ruthenium red, indicating reduced food intake by the agonists. In a very recent study, it was observed that co-treatment with dietary TRPA1 agonist, allicin rich garlic juice was able to reverse increased food intake, body weight, impaired glucose homeostasis, elevated PYY, ghrelin and decreased GLP-1, CCK produced by high fat-diet (102).

Another major event serving a critical part in appetite regulation is GI inflammation. As TRPA1 channels are present on capsaicin-sensitive sensory neurons, they have also shown involvement in inflammatory bowel disease (IBD) along with TRPV1 channels. Patients suffering from ulcerative colitis and Crohn’s disease have shown upregulation in the expression of TRPA1 and TRPV1 mRNA, although results for TRPV1 are contradictory (103). TRPA1 antagonists, as well as knockdown, supposedly help in decreasing colitis severity and reversing visceromotor response in mice (104), but studies have also suggested the opposite (105). Lastly, TRPA1 channels are also involved in olfaction. The sense of smell is essential for stimulation of appetite and further release of gastric hormones for digestion of food. TRPA1 channels mediate nociception conjured by pungent substances. TRPA1 knockout mice are unable to sense foul odors and thus fail to avoid the chamber filled with formalin, acrolein or AITC whereas the wild type animals show avoidance behavior (106).

### TRPA1 in the Hypothalamic Regulation of Appetite

Not many studies have been conducted to confirm the presence of TRPA1 channels in the hypothalamus (mainly in the supraoptic nucleus) (39, 107), but the supporting evidence is convincing enough to say that TRPA1 channels could exist in the arcuate nucleus of the hypothalamus where they temper various appetite-controlling neuropeptides to modulate hunger and food intake. Moreover, as mentioned earlier, TRPV1 and TRPA1 are closely related in expression and functions; several studies have affirmed the presence of TRPV1 channels in the arcuate nucleus (20, 80). A study using TRPA1 agonist phenethyl isothiocyanate revealed enhanced mRNA expression of anorexigenic peptide POMC and reduced NPY/AgRP expression, which is orexigenic in nature, thus plummeting food intake in mice (108). Cinnamaldehyde supplementation also proved to enhance mRNA expression of anorectic peptides such as POMC, CART, and CCK in the hypothalamus of high-fat diet-fed mice (58). Vomitoxin (deoxynivalenol), which occurs as mycotoxin in grains such as wheat, oats, rice and barley, has been reported to induce TRPA1 mediated calcium signaling in murine STC-1 cell lines (109). *In vivo* study in mice showed a significant increase in POMC, CART and melanocortin four receptor (MC4R) mRNA expression in the arcuate nucleus of the hypothalamus after vomitoxin treatment and also leads to reduced food intake during the dark phase (110). Tominaga et al. (2016) also reported increased expression of hypothalamic POMC, MC4R, gastric CCK expression and TRPA1 expression in mice after vomitoxin treatment hinting that vomitoxin exerts is anorexic effects by increasing hypothalamic anorexic peptides *via* TRPA1 activation (111).

Regardless of numerous studies suggesting a positive role of TRPA1 agonists controlling appetite and metabolism, some studies tend to differ as well. A study in Wistar rats reported enhanced ghrelin levels in the blood and heightened food intake after treatment with TRPA1 agonist β-eudesmol, while TRPA1 knockout animals showed standard food intake. It also increased histamine levels in the hypothalamic tuberomammillary nucleus and elevates gastric vagal nerve activity (GVNA) responsible for the absorption and digestion of nutrients. However, pretreatment with HC-030031 eliminated the elevation in GVNA (112).

## Conclusion

**Figure 1:**
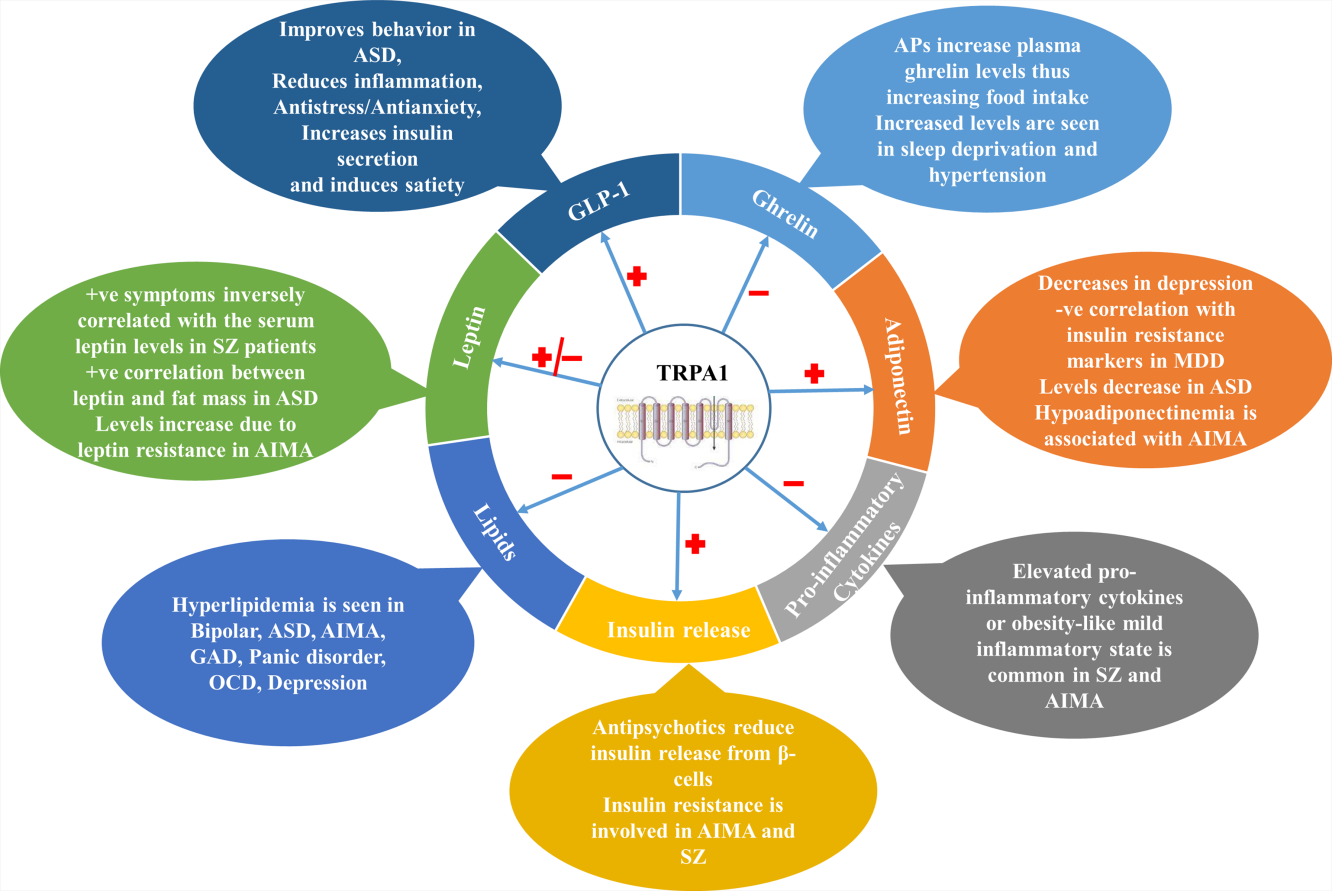
Involvement of TRPA1 channels in various NPDs through different metabolic markers. TRPA1 channels modulate various metabolic parameters such as Ghrelin, Leptin, GLP-1, Adiponectin in the body which are involved in the pathophysiology of various NPDs. They also regulate insulin release from pancreatic β-cells, pro-inflammatory cytokine release and lipid balance in the body. ASD, Autism Spectrum Disorder; APs, Antipsychotics; AIMA, Antipsychotics-induced metabolic alterations; MDD, Major Depressive Disorder; GAD, General Anxiety Disorder; OCD, Obsessive Compulsive Disorder; GLP-1, Glucagon-like Peptide-1; TRPA1, Transient Receptor Potential Channel Ankyrin 1; SZ, Schizophrenia.

Metabolic comorbidities like T2DM, obesity and cardiovascular disorders have been greatly associated with NPDs, and are responsible for reduced life expectancy and poor quality of life in the patients. Along with the co-existence, NPDs and metabolic disorders also share a commonality in pathophysiological changes like appetite dysregulation, altered lipid and carbohydrate homeostasis, heightened inflammatory responses and altered adipokine-cytokine levels. TRPA1 channels, which are distributed throughout the body, are emerging as potential therapeutic targets for metabolic disorders and NPDs. TRPA1 channels have been found to play a cardinal role in NPDs (like depression and anxiety), Alzheimer’s disease, Huntington’s disease, multiple sclerosis, migraine, and age-related cognitive impairment in different rodent models. Parallelly, TRPA1 channels are found to be playing a pivotal role in metabolic alterations like diet-induced obesity, appetite dysregulation, adiposity, inflammatory response, and regulation of peripheral appetite-regulating peptides (like insulin, leptin and ghrelin) release and action as depicted in **Figure 1**.

Taking all together, modulation of central as well as peripheral TRPA1 channels can be a novel therapeutic strategy to protect against metabolic adversities associated with NPDs, out of which the major ones are weight gain and diabetes. Controlling appetite with natural TRPA1 modulators could help improve patient compliance in NPDs and normal individual health and well-being. Consequently, further studies involving specific TRPA1 modulators need to be designed to target metabolic syndrome involving NPDs.

## Author Contributions

RKS and RS: Conceptualization, Writing- original draft preparation. YB: Writing- original draft preparation. MB and IP: Writing- Reviewing and Editing. TS and AK: Writing- Reviewing and Editing, Visualization, Supervision, Project administration. All authors contributed to the article and approved the submitted version.

## Conflict of Interest

The authors declare that the research was conducted in the absence of any commercial or financial relationships that could be construed as a potential conflict of interest.

## Publisher’s Note

All claims expressed in this article are solely those of the authors and do not necessarily represent those of their affiliated organizations, or those of the publisher, the editors and the reviewers. Any product that may be evaluated in this article, or claim that may be made by its manufacturer, is not guaranteed or endorsed by the publisher.
